# Rituximab retreatment guided by CD27+ B-cell count vs. clinical relapse in anti-MAG polyneuropathy: a cost-effective approach with lower cumulative doses

**DOI:** 10.3389/fneur.2025.1659670

**Published:** 2025-09-15

**Authors:** Margherita Bellucci, Giovanna Capodivento, Federico Massa, Federica Bozzano, Giacomo Bavestrello, Elena Baroncelli, Corrado Cabona, Antonia Cagnetta, Angelo Schenone, Lucilla Nobbio, Luana Benedetti

**Affiliations:** ^1^Department of Neuroscience, Rehabilitation, Ophthalmology, Genetics, Maternal and Child Health (DINOGMI), University of Genova, Genoa, Italy; ^2^IRCCS Ospedale Policlinico San Martino, Genoa, Italy; ^3^Division of Clinical Neurophysiology and Epilepsy Center, IRCCS Ospedale Policlinico San Martino, Genoa, Italy; ^4^Clinic of Hematology, Department of Internal Medicine and Medical Specialties (DiMI), University of Genoa, Genoa, Italy

**Keywords:** anti-MAG polyneuropathy, rituximab maintenance therapy, B-cell depletion, chronic inflammatory neuropathy treatment protocols, CD27 B cells monitoring

## Abstract

**Introduction:**

Rituximab (RTX) is a widely used treatment for anti-MAG polyneuropathy, though standardized maintenance strategies are lacking. We aimed to compare two RTX retreatment protocols: (1) a full course (375 mg/m^2^/week for 4 weeks) administered at clinical relapse, and (2) a single infusion (375 mg/m^2^) at reappearance of peripheral CD27+ B cells—to evaluate their impact on disability progression over time.

**Patients and methods:**

We retrospectively enrolled 29 patients with anti-MAG polyneuropathy, dividing them into two cohorts: (1) *relapse* (*n* = 19), treated with a full course at clinical relapse, or (2) *Kim's protocol* (*n* = 10), treated based on peripheral CD27+ B cell monitoring. Changes in INCAT, MRC sum score, and ISS from baseline to last follow-up were assessed.

**Results and discussion:**

No significant changes in MRC scores were observed in either cohort. Both cohorts showed a significant reduction in INCAT scores at last follow-up, with a tendency toward greater improvement in *Kim's protocol* cohort. ISS scores were significantly lower in *Kim's protocol* cohort compared to the *relapse* cohort (*p* < 0.01). Importantly, patients treated according to Kim's protocol received a cumulative RTX dose ~2.5 times lower than those treated upon relapse (*p* < 0.0001), despite showing comparable or better clinical outcomes.

**Conclusion:**

A tailored maintenance strategy guided by peripheral CD27+ memory B-cell monitoring enables reduced cumulative RTX exposure while preserving clinical efficacy. This approach may improve cost-effectiveness and reduce treatment burden in patients with anti-MAG polyneuropathy.

## 1 Introduction

Anti-myelin-associated glycoprotein (MAG) antibody neuropathy is a chronic demyelinating polyneuropathy characterized by both a progressive, distal- and sensory-predominant impairment with postural tremor in the upper limbs and gait sensory ataxia; motor involvement and disability usually occur later during disease progression.

Nerve conduction studies display sensory abnormalities consistent with demyelination, namely prevalent distal nerve conduction slowing, and abnormally increased latencies (1).

Anti-MAG polyneuropathy is the most common paraproteinemic IgM neuropathy frequently associated with an IgM monoclonal gammopathy of undetermined significance (MGUS); however, it can be also related to a lymphoproliferative condition (2–4).

To date, satisfactory immunotherapy is not available for the treatment of patients with anti-MAG neuropathy (5, 6). Regardless of the underlying haematologic condition, however, the literature indicates that at least 60% of individuals with anti-MAG neuropathy respond to rituximab (RTX), an anti-CD20 monoclonal antibody (7–9). Indeed, a 2016 Cochrane review, analyzing all randomized controlled trials (RCTs) in which RTX was used, displayed a potential efficacy of this drug in paraproteinemic neuropathies, albeit with weak evidence (5).

The efficacy of rituximab in anti-MAG polyneuropathy has been supported by previous studies, suggesting a typically prolonged therapeutic response, with clinical relapses generally not occurring before 2 years after initial treatment, though they may present beyond this period and the precise timing remains variable (8, 10, 11).

However, no standardized long-term treatment protocol for RTX has been defined yet. In fact, different maintenance schedules exist that are adopted by clinicians based on personal expertise rather than guided by evidence. These include the infusion of 1 g of RTX every 6 months (12, 13), or administration of a full course (375 mg/m^2^/week for 4 weeks) at clinical relapse (8). However, maintenance treatment schedules and timing are still debated (11, 13). Retreating strategy could follow the reemergence of peripheral CD27+ memory B cells, similarly to the treatment scheme of Neuromyelitis Optica Spectrum Disorder (NMOSD) (14, 15). According to this protocol, following a cycle of induction therapy, a single maintenance infusion of RTX (375 mg/m^2^) was administered whenever the frequency of reemerging CD27+ memory B cells in peripheral blood mononuclear cells, measured by flow cytometry, exceeded 0.05% in the first 2 years and 0.1% thereafter. In NMOSD this tailored dosing regimen has been demonstrated to determine a positive long-lasting clinical response.

We translated the treatment strategy used in NMOSD to anti-MAG neuropathy based on their biosimilarity as B-cell–mediated autoimmune diseases driven by pathogenic autoantibodies. Indeed, both disorders involve CD27+ memory B cells, which play a key role in disease activity and serve as biomarkers for monitoring therapeutic response. RTX effectively targets B cells in both conditions, supporting the rationale for CD27+ B-cell monitoring in anti-MAG neuropathy, similarly to its well-established use in NMOSD (11, 16–19).

However, long-term immunomodulatory therapy with RTX in immune-mediated neuropathies is associated with a significant burden of side effects—particularly infectious complications—alongside notable treatment costs and substantial logistical demands, including regular infusions and intensive healthcare coordination for both patients and infusion centers (20–23).

In this study we compare the long-term effect of two distinct retreatment strategies with RTX adopted in our center, namely (i) a full course (375 mg/m^2^/week × 4 weeks) at every clinical relapse, or (ii) a single maintenance infusion (375 mg/m^2^) at the reappearance of peripheral CD27+ B cells.

The aim of our study was to determine whether patients treated with RTX at clinical relapse experienced an accumulation of disability in the long term compared to those treated on a periodic basis according to their peripheral immunophenotype, regardless of the disease's clinical expression.

## 2 Materials and methods

### 2.1 Patients and study design

We retrospectively included patients with anti-MAG neuropathy responding to a first course of RTX at a dose of 375 /mg/m^2^ per week for 4 weeks.

All patients underwent nerve conduction studies (NCS) and electromyography (EMG) at diagnosis, which revealed a demyelinating pattern with diminished sensory nerve action potentials and delayed distal motor latencies, consistent with typical anti-MAG neuropathy (1). No axonal or normal NCS/EMG patterns were observed. Other potential causes of neuropathy were excluded based on a comprehensive clinical and laboratory evaluation, which included negative screening for metabolic, toxic, nutritional, and paraneoplastic etiologies.

The INCAT disability scale (24), Medical Research Council (MRC) sum score (25), and ISS (INCAT Sensory sum Score) (26) clinical scales were administered at baseline (pre-RTX), and then every 6 months, until the last follow-up (FU) visit. Response to therapy was defined as an improvement of at least one point in two out of the three aforementioned clinical scales at 12-month timepoint (22, 23).

Patients were followed from January 1, 2003, to June 31, 2024, and divided into two cohorts according to the RTX re-treatment schedule used as maintenance therapy:

1) *Relapse*, when they were treated with a complete course (i.e., 375 mg/m^2^/week χ 4 weeks) at every clinical relapse; relapse was defined as a ≥2-point decrease in MRC sum score, ≥1-point increase in INCAT disability score, or ≥2-point increase in ISS (29, 30).
2) *Kim's protocol*, when they were treated with a single infusion (i.e., 375 mg/m^2^) based on peripheral CD27+ B lymphocytes. RTX was administered whenever the frequency of reemerging CD27+ memory B cells in peripheral blood mononuclear cells, exceeded 0.05% in the first 2 years and 0.1% thereafter (14, 15).

Patients were assigned to the two cohorts based on distinct time frames, which reflected the timing of the adoption of Kim's treatment protocol at our center. Specifically, patients managed between 2003 and 2011—prior to the implementation of the Kim protocol—were assigned to the *relapse* cohort, whereas those treated from 2012 to 2024 were included in *Kim's protocol* cohort.

We assessed the number of full RTX courses or single infusions received by each patient, and the time passed between RTX administration and CD27+ reoccurrence in patients treated according to Kim's protocol.

Furthermore, we collected the presence or absence of distal neuropathic tremor as well as safety data, namely the occurrence of RTX-related adverse events after infusion, including hypogammaglobulinemia.

### 2.2 Laboratory assessment

Anti-MAG IgM antibody titer was assessed pre-RTX and at the last FU using a commercial ELISA kit, and Bühlmann titer units or BTUs were used to express the results (Bühlmann, Schönenbuch, Switzerland).

Cytofluorometry was used to analyze peripheral immunophenotype in patients treated based on reemerging CD27+ memory B cells. To ascertain whether treatment was necessary, sampling was done at each subsequent scheduled medical appointment following RTX administration, typically every 6 months.

### 2.3 Statistical analysis

We compared demographical, clinical data (i.e., INCAT disability scale, MRC sum score, and ISS, tremor presence), and anti-MAG antibody titer between pre-RTX and the FU timepoints, using the two-tailed Student's *t*-test or Wilcoxon rank-sum test based on normal distribution. The same variables were compared between the relapse and Kim's protocol cohorts.

To explore a potential relationship between baseline anti-MAG antibody titers and CD27+ cell re-appearance dynamics, we performed a subgroup analysis within the *Kim's protocol* cohort. Patients were stratified into two subgroups based on their baseline anti-MAG titers: *high-* (>70,000 BTU) or *low-titer* (<700,000 BTU) (31). As CD27+ cell counts were used to guide timing of retreatment, the interval between RTX maintenance infusions was used as a proxy of memory *B*-cell reconstitution. Mean retreatment intervals were compared between the two groups using an unpaired *t*-test.

Statistical differences were significant when *p* < 0.05. GraphPad Prism software version 9 (GraphPad Software Inc., California, USA) was used to perform all the statistical comparisons and to generate most of the graphs.

## 3 Results

### 3.1 Demographical and clinical data

Demographic and clinical features of patients included in the analyses are displayed in Tables 1–3. We analyzed data from 29 patients, 9 females and 20 males. The mean age was 67.3 years (±SD 10.46; range: 48–83) at disease onset, and 75.5 years at last FU (±9.28; range: 55–89). The mean FU duration was 8.1 years (±5.81; range: 2–20). However, FU time varied significantly between groups, being 4.20 (±3.16; 2–12) years in the *Kim's protocol* cohort and 10.26 (±5.84; 2–20) years in the *relapse* cohort (*p* = 0.002) due to the distinct enrollment time frames.

**Table 1:** Demographical and clinical data.

**Patient^#^**	**Age at onset (years)–Sex**	**Follow-up duration (years)**	**Tremor**	**MRC sum score (pre RTX–last FU)**	**ISS score (pre RTX–last FU)**	**INCAT (pre RTX–last FU)**	**Anti-MAG Ab (BTU) (pre RTX–last FU)**	**Haematologic disease**	**IgM levels (g/L) (pre RTX–last FU)**	**Maintenance therapy cohort^#^**	Number of RTX maintenance doses (^*^vs. §)
1	78–F	7	Present	60–58	3–4	2–4	162,506–16,413	WM	11.5–0.5	Relapse	3^*^
2	55–F	8	Absent	60–60	8–3	1–1	49,694–12,801	MGUS IgM	5.2–9.6	Relapse	1^*^
3	57–M	8	Present	53–53	8–9	3–2	NA−34,544	CLS	2.8–0.9	Relapse	4^*^
4	65–M	7	Present	58–56	3–4	2–1	59,738–229,300	MGUS IgM	5.6–4.6	Relapse	2^*^
5	85–F	2	Absent	60–60	3–3	3–2	14,988–NA	WM	7.6–NA	Relapse	2^*^
6	70–M	7	Present	60–60	7–3	2–2	409,000–33,396	MGUS IgM	5.6–3.9	Relapse	2^*^
7	73–M	6	Present	53–52,5	4–3	4–3	51,009–86,650	WM	5.9–3.1	Relapse	4^*^
8	48–M	8	Present	60–60	3–2	3–2	53,630–279,700	WM	5.1–2.1	Relapse	2^*^
9	51–M	4	Present	57–60	4–6	4-3	66,865–371,400	MGUS IgM	3.6–2.0	Relapse	3^*^
10	57–M	9	Absent	54–54	3–3	2-2	60,000–60,648	WM	0.6–1.9	Relapse	3^*^
11	78–M	11	Absent	60–60	6–6	1–1	50,288–75,100	CLL	2.7–2.5	Relapse	3^*^
12	53–F	20	Absent	58–58	6–4	5–3	NA−10,917	MGUS IgM	2.9–0.8	Relapse	3^*^
13	65–M	20	Absent	58–53	8–4	4–3	26,931–34,776	WM	10.9–15.4	Relapse	3^*^
14	73–M	2	Present	58–60	9–2	5–3	250,000–123,200	WM	NA–NA	Relapse	2^*^
15	59–F	20	Absent	51,5–53	5–6	5–4	50,420–71,916	MGUS IgM	3.8–NA	Relapse	5^*^
16	66–M	14	Absent	59–51	3–6	3–5	NA−170,667	MGUS IgM	4.3–2.1	Relapse	2^*^
17	74–M	14	Present	58–60	12–12	4–3	153140–NA	MGUS IgM	4.2–3.5	Relapse	1^*^
18	60–M	18	Absent	58–60	8–5	3–1	51,200–8,479	MGUS IgG	4.7–3.9	Relapse	2^*^
19	50–F	10	Present	46 – 48.5	7–4	5–4	483,776–56,000	MGUS IgM	1.9–1.4	Relapse	4^*^
20	81–M	2	Present	60–60	4–1	3–2	299,000–51,186	CLS	4.3–NA	Kim	1^§^
21	70–F	4	Absent	60–60	2–1	1–0	235,000–NA	MGUS IgM	3.0–1.8	Kim	1^§^
22	66–M	7	Present	60–60	4–2	1–0	100,700–43,000	WM	4.4–0.6	Kim	2^§^
23	80–M	2	Absent	55–60	3–2	4–2	4,219–1,514	NHL	2.5–1.6	Kim	1^§^
24	83–M	2	Present	60–60	3–1	2–1	467,000–99630	MGUS IgM	5.8–2.4	Kim	1^§^
25	69–M	12	Absent	60–60	6–3	2–2	10,812–9,292	MGUS IgG	4.6–2.6	Kim	8^§^
26	66–F	4	Present	55–60	7–1	5–3	65,000–18,961	MGUS IgM	2.6–1.6	Kim	2^§^
27	78–M	3	Present	58–56	4–3	2–2	54,430–19,135	MGUS IgM	NA–NA	Kim	2^§^
28	70–F	4	Absent	60–60	8–5	3–2	56,068–30,640	MGUS IgM	1.5–1.1	Kim	1^§^
29	73–M	2	Absent	52.5–57	10–4	5–4	14,786–21,640	MGUS IgM	6.5–NA	Kim	1^§^

Ab, antibodies; BTU, Bühlman titre unit; CLL, chronic lymphatic leukemia; CLS, chronic lymphoproliferative syndrome; M, male; F, female; FU, follow-up; ISS, INCAT sensorysum score; INCAT, inflammatory neuropathy cause and treatment; MAG, myelin-associated-glycoprotein; MGUS, monoclonal gammopathy of undetermined significance; MRC, medical research sum; NA, non-available; NHL, Non Hodgkin lymphoma; RTX, rituximab; WM, Waldenström macroglobulinemia.

^§^rituximab single infusion (375 mg/m^2^); ^*^rituximab full course (375 mg/m^2^/week × 4 weeks).

^#^Maintenance therapy cohort: Kim, patients treated with a single RTX infusion (375 mg/m^2^) based on peripheral CD27+ B lymphocytes; relapse, patients treated with a full course of RTX (375 mg/m^2^/week × 4 weeks) following clinical deterioration.

**Table 2:** Comparison of demographical and clinical data in the two cohorts.

**Patient characteristics**	**All patients (*n* = 29)**	**Relapse cohort (*n* = 19)**	**Kim's protocol cohort (*n* = 10)**	***p* value**
Age at onset (years)	67.34 ± 10.46 (48–85)	64.05 ± 10.80 (48-85)	73.60 ± 6.38 (66-83)	0.006
Sex	9 F 20 M	6 F 13 M	3 F 7 M	NS
Follow-up duration (years)	8.10 ± 5.81 (2–20)	10.26 ± 5.84 (2–20)	4.20 ± 3.16 (2–12)	0.002
Symptoms duration before treatment (months)	8.07 ± 3.06 (3–15)	8.37 ± 3.85 (3–15)	7.50 ± 2.55 (4–12)	NS
Presence of tremor (*n* =)	15	10	5	NS
MRC sum score at baseline	57.31 ± 3.46 (46–60)	56.92 ± 3.77 (46–60)	58.05 ± 2.83 (52.5–60)	NS
ISS score at baseline	5.55 ± 2.58 (2–12)	5.79 ± 2.63 (3–12)	5.10 ± 2.55 (2–10)	NS
INCAT at baseline	3.07 ± 1.36 (1–5)	3.21 ± 1.31 (1–5)	2.80 ± 1.48 (1–5)	NS
Anti-MAG Ab (BTU) at baseline	126,930 ± 142,345 (4,219–483,776)	130,732 ± 146,843 (14,988–483,776)	119,113 ± 158,360 (4,219–467,000)	NS
IgM (g/L)	4.61 ± 2.50 (0.61–11.52)	4.95 ± 2.81 (0.61–11.52)	3.94 ± 1.65 (1.51–6.54)	NS
Haematologic disease (*n* =)	15 MGUS IgM 8 WM 2 MGUS IgG 2 CLS 1 NHL 1 CLL	9 MGUS IgM 7 WM 1 MGUS IgG 1 CLS 1 CLL	6 MGUS IgM 1 WM 1 MGUS IgG 1 CLS 1 NHL	NS

Data are expressed as mean ± standard deviation (SD) and range (values between parentheses).

Ab, antibodies; BTU, Bühlman titre unit; CLL, chronic lymphatic leukemia; CLS, chronic lymphoproliferative syndrome; M, male; F, female; ISS, INCAT sensorysum score; INCAT, inflammatory neuropathy cause and treatment; MAG, myelin-associated-glycoprotein; MGUS, monoclonal gammopathy of undetermined significance; MRC, medical research sum; n, number of patients; NS, non-significant; NHL, non hodgkin lymphoma; WM, Waldenström macroglobulinemia.

**Table 3:** Clinical scales scores and comparison at the last follow-up.

**Clinical scores**	***Relapse* cohort (*n* = 19)**	***Kim's protocol* cohort (*n* = 10)**	***p* value**
MRC sum score pre-RTX	56.92 ± 3.77 (55.10–58.74)	58.05 ± 2.83 (56.02–60.08)	NS
MRC sum score last FU	56.68 ± 3.86 (54.83–58.54)	59.30 ± 1.49 (58.23–60.37)	*p* = 0.0501
INCAT pre-RTX	3.21 ± 1.31 (2.58–3.85)	2.80 ± 1.48 (1.74–3.86)	NS
INCAT last FU	2.58 ± 1.17 (2.02–3.14)	1.80 ± 1.23 (0.92–2.68)	NS
ISS score pre-RTX	5.79 ± 2.63 (4.51–7.06)	5.10 ± 2.55 (3.27–6.93)	NS
ISS score last FU	4.68 ± 2.47 (3.49–5.88)	2.30 ± 1.41 (1.28–3.31)	^**^p < 0.01
Anti-MAG Ab (BTU) pre-RTX	130,732 ± 146,843 (45,577–215,146)	119,113 ± 158,360 (12,614–240,839)	NS
Anti-MAG Ab (BTU) last FU	104,270 ± 110,829 (40,279–168,261)	32,778 ± 29,469 (10,126–55,429)	*p* = 0.0743

Table shows clinical scales scores at baseline (pre-RTX) and at last follow-up (FU); *p*-value is referring to the comparison between the two cohorts at the most recent FU.

Data are expressed as mean ± standard deviation (SD) and confidence interval (CI) (values between parentheses). NS, not significant. ^**^p < 0.01.

Concerning related hematological disorders, 15 patients had MGUS IgM (51.7%), 8 had Waldenström's Macroglobulinemia (27.6%), 2 had Chronic Lymphoproliferative Syndrome consistent with a low-grade B-cell lymphoproliferative disorder (6.9%), 2 had MGUS IgG (6.9%), 1 had non-Hodgkin lymphoma (3.4%), and 1 had Chronic Lymphatic Leukemia (3.4%). The clinical course of underlying hematological conditions was stable throughout the FU, with no treatment escalation required. Indeed, RTX was the only treatment administered, for both the neuropathy and hematological conditions.

Tremor was present pre-RTX in 15 out of 29 patients (51.7%); in particular, tremor was detected in 10 out of 20 patients in the *relapse* cohort (50%) and in 5 out of 10 patients in the *Kim's protocol* cohort (50%).

The mean baseline scores obtained at MRC, ISS and INCAT were 57.3 (±3.5), 5.6 (±2.6), and 3.1 (±1.4), respectively.

Nineteen (*n* = 19) patients were included in the *relapse* cohort, and ten patients (*n* = 10) were included in the *Kim's protocol* cohort.

**Figure 1:**
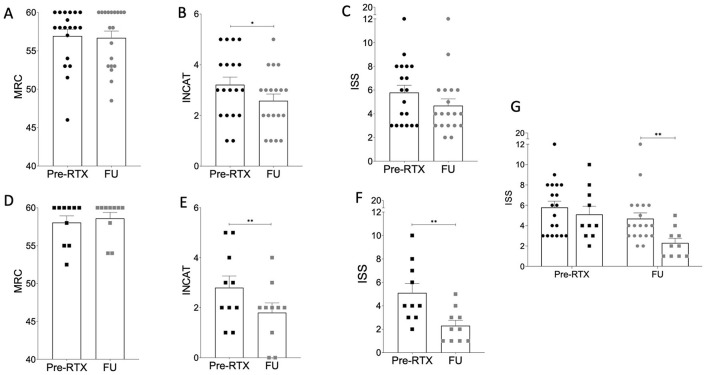
Scores obtained on scales in the cohort of patients treated upon relapse **(A–C)** and according to Kim's protocol **(D–F)**. MRC, INCAT and ISS were evaluated before RTX treatment (pre-RTX) and at the last follow-up (FU). In the relapse cohort, only the INCAT score appears to be improving at FU **(B)**. In the Kim's protocol cohort, both INCAT and ISS scores improve at FU **(E, F)**. Lastly, when comparing last FU, the ISS scores in the Kim's protocol cohort appear significantly lower than those of the relapse one **(G)**. Paired two-tailed *T*-test was used for statistical comparison between pre-RTX treatment and FU in **(A–F)**. In **(G)** unpaired multiple comparison was used. *p < 0.05; **p < 0.01.

Due to the different enrollment time frames, patients in the *Kim's protocol* cohort were older at disease onset and had a significantly shorter follow-up compared to those in the relapse cohort. The older age in the *Kim's protocol* cohort was not a determinant in treatment selection, but rather a consequence of the more recent adoption of this treatment strategy, which may have influenced the demographic profile of this subgroup. Despite these differences, the two cohorts were clinically comparable at baseline in terms of neurological disability and clinical scale scores (Figure 1, Table 2).

**Figure 2:**
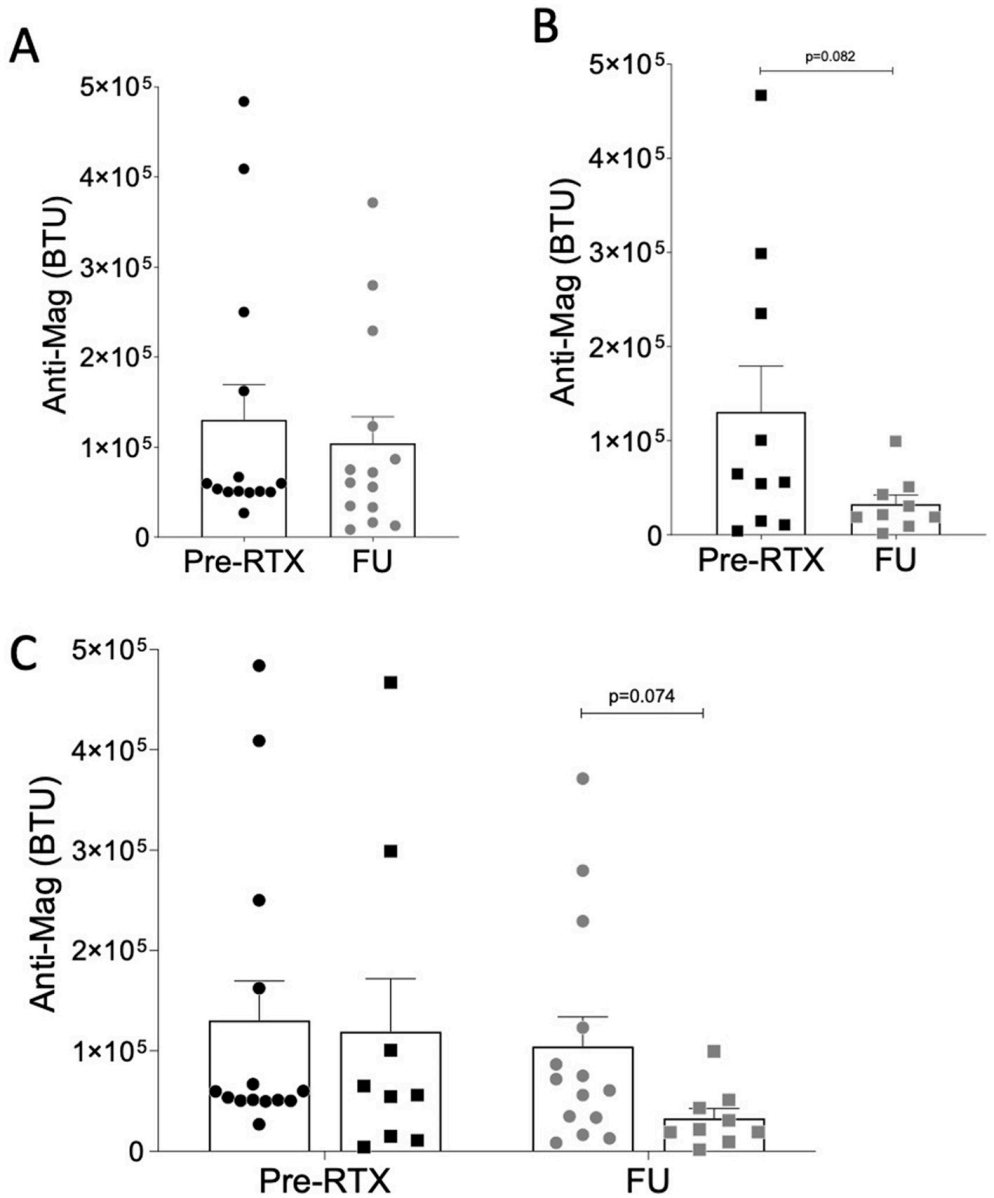
Anti-MAG antibody titer (BTU) in the relapse cohort **(A)** and in Kim's protocol cohort **(B)**. Paired two-tailed *T*-test was used for statistical comparison between pre-RTX treatment and FU **(A, B)**. In **(C)** unpaired multiple comparison was used.

Similarly, the comparison of anti-MAG titer between the two cohorts pre-RTX did not yield any significant statistical difference (Figure 2).

### 3.2 Clinical outcomes

#### 3.2.1 MRC sum score

In the *relapse* cohort, the comparison of MRC scale scores pre-RTX and at the FU timepoints (mean ± SD: 56.92 ± 3.77 vs. 56.68 ± 3.86; Table 2; Figure 1) did not demonstrate a statistically significant difference. Similarly, in the *Kim's protocol cohort* we did not observe any significant reduction of MRC scores from pre-RTX to FU (58.05 ± 2.83 vs. 59.30 ± 1.49; Table 2; Figure 1). When comparing the two cohorts at the most recent FU, the reduction in MRC score observed in the *Kim's protocol* cohort showed a trend toward statistical significance compared to the *relapse* cohort (*p* = 0.0501; Table 2).

#### 3.2.2 INCAT disability scale

In the *relapse* cohort we observed a significant reduction in INCAT scores at the FU timepoint compared to the pre-RTX scores (2.58 ± 1.17 vs. 3.21 ± 1.31; *p* < 0.05; Table 2; Figure 1).

Furthermore, we also found a substantial decrease in INCAT scores in the *Kim's protocol* cohort at the most recent follow-up (2.80 ± 1.48 vs. 1.80 ± 1.23; *p* < 0.01; Table 2; Figure 1) as compared to the pre-RTX scores. The comparison between the two cohorts at the most recent FU did not reveal any statistically significant differences (Table 2).

#### 3.2.3 ISS

We did not find significant variations in ISS values between pre-RTX and the FU timepoint (5.79 ± 2.63 vs. 4.68 ± 2.47; Table 2; Figure 1) in the *relapse* cohort.

Conversely, we observed a substantial decrease in the ISS score at the most recent FU in the *Kim's protocol* cohort as compared to the pre-RTX evaluation (2.30 ± 1.41 vs. 5.10 ± 2.55; *p* < 0.01; Table 2; Figure 1).

Of note, when comparing the two cohorts at the most recent FU, the ISS scores in the *Kim's protocol* cohort were significantly lower than those of the *relapse* cohort (*p* < 0.01; Table 2; Figure 1).

#### 3.2.4 Anti-MAG antibodies

Anti-MAG antibody titer was available from 14 out of 19 patients in the *relapse* cohort, and from 9 out of 10 patients in the *Kim's protocol* cohort.

We did not find statistically significant difference in serum anti-MAG levels between pre-RTX and last FU (130,732 ± 146,843 vs. 104,270 ± 110,829; Table 2; Figure 2) in the *relapse* cohort.

In the *Kim's protocol* cohort, serum anti-MAG levels at the last FU were numerically lower compared to pre-RTX values (119,113 ± 158,360 vs. 32,778 ± 29,469), although this difference did not reach statistical significance (*p* = 0.083; Table 3; Figure 2)

When the two cohorts are solely compared at the last FU, a *p*-value of 0.074 (Table 2; Figure 2) suggests a numerically greater reduction in antibody titer in the *Kim's protocol* cohort than in the *relapse* one, albeit not reaching statistical significance.

Lastly, we investigated a possible indirect association between anti-MAG titers and CD27+ cell re-emergence by analyzing the mean interval between RTX infusions in the two subgroups of the *Kim's protocol* cohort, stratified by baseline antibody levels (*high-titer* >70,000 BTU vs. *low-titer* < 700,000 BTU). The mean interval was 16.6 months (±8.4) in the *high-titer* cohort and 14.3 months (±6.4) in the *low-titer* cohort, with no statistically significant difference (*p* = 0.596).

#### 3.2.5 Tremor

Thirteen out of 15 patients (86.7%) who had tremor at baseline achieved improvement at the last follow-up in both treatment cohorts. Tremor failed to improve only in two patients (13.3%), both included in the *relapse* cohort.

### 3.3 Rituximab administration

**Figure 3:**
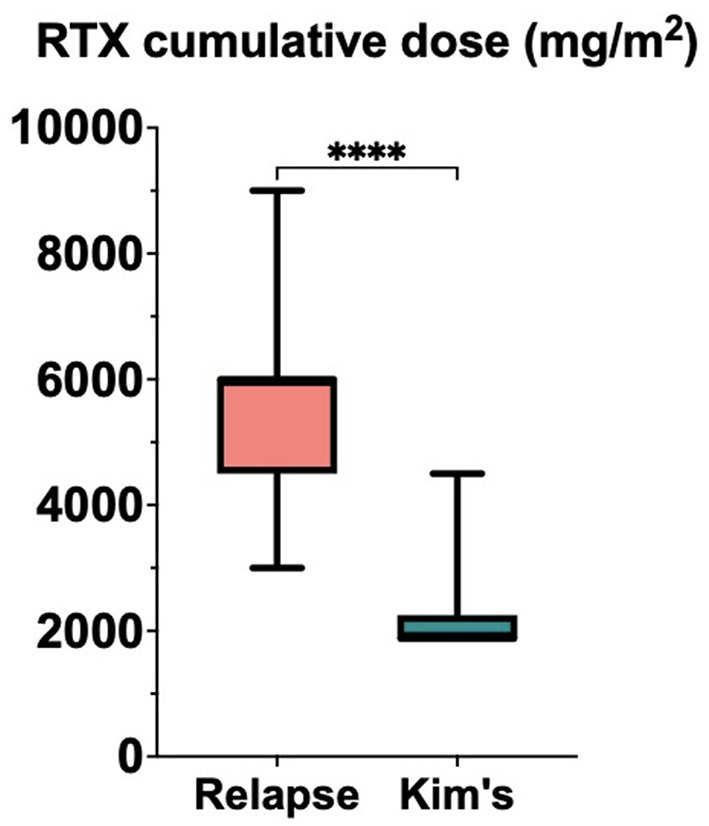
Box plot illustrating the cumulative rituximab (RTX) dose (mg/m^2^) in the relapse cohort and Kim's protocol cohort. The cumulative dose was significantly lower in the Kim's protocol cohort, ****p < 0.0001.

During follow-up, patients in the *relapse* cohort received a mean of 2.7 complete cycles of RTX (375 mg/m^2^/week x 4 weeks) (SD ± 1.10; range 1–5), corresponding to a mean cumulative dose of 5,526.32 mg/m^2^ per patient (±1,585.30). In contrast, patients in the *Kim's protocol* cohort received a mean of 2 single maintenance infusions (375 mg/m^2^) (±2.16; range 2–8), with a mean cumulative dose of 2,250 mg/m^2^ per patient (±810.10). A comparison of cumulative RTX exposure demonstrated a significantly lower total dose in the *Kim's protocol* cohort relative to the *relapse* cohort (*p* < 0.0001), as illustrated in Figure 3.

One patient in the *relapse* cohort became a non-responder after the second course of RTX (2 years of disease), while in the *Kim's protocol* cohort one patient stopped responding to rituximab after receiving 7 maintenance infusions (11 years of disease).

Regarding RTX safety, only one patient experienced a mild infusion reaction, which did not require the discontinuation of the treatment. No patient developed hypogammaglobulinemia.

### 3.4 Reemerging CD27+ memory B cells

In *Kim's protocol* cohort, the average time between RTX infusion and reappearance of CD27+ memory B cells above the established cut-off was 14.9 months (±6.7), with a range of 6–31 months.

### 3.5 *Post-hoc* analysis

To address concerns related to the small sample size (*n* = 29), we performed a *post hoc* power analysis on the primary outcome, which showed a statistically significant difference between groups: the ISS score at last follow-up. Based on the observed means and standard deviations (2.30 ± 1.41 in the *Kim's protocol* cohort vs. 4.68 ± 2.47 in the *relapse* cohort), the estimated effect size (Cohen's *d*) was approximately 1.09. Given the actual group sizes (*n* = 10 and *n* = 19) and a two-tailed alpha level of 0.05, the calculated statistical power was approximately 77%. Although slightly below the conventional 80% threshold, this level of power is generally considered acceptable in retrospective exploratory studies, especially in the context of rare diseases such as anti-MAG neuropathy. These findings support the robustness of the observed between-group difference in ISS scores despite the limited sample size.

## 4 Discussion

In our research, the tailored treatment plan based on periodic CD27 lymphocyte monitoring showed an improvement in sensory impairment, and a considerable reduction in the total amount of RTX administered as compared to treatment upon clinical relapse.

Our results disclose a significant reduction in ISS scores at last follow-up in the cohort of patients treated according to Kim's protocol. Since distal sensory impairment is the most prevalent manifestation of anti-MAG neuropathy, this result is especially valuable, with the persistence of improvement over the time highlighting a significant therapeutic impact of this RTX regimen.

We did not find a significant difference between the two treatment protocols when the MRC and INCAT scores were compared. This might be determined by the inadequacy of these outcome measures, as already pointed out in previous RCTs (27, 28). Given that MRC scale does not account for the distal motor sectors that are most affected in anti-MAG neuropathy, and since its expression is typically sensory dominant, it may not accurately reflect clinical impairment in this condition. Of note, the MRC score at baseline was at the maximum (60/60 points) in 12 out of 29 patients, which makes challenging to eventually appreciate a clinical variation in this domain. Concerning the INCAT scores, both treatment protocols achieved a satisfactory clinical response to RTX. However, the reduction in disability appeared numerically slightly more pronounced in the *Kim's protocol* cohort, although this difference did not reach statistical significance and should be interpreted with caution due to the small sample size and the retrospective nature of the study. Similarly, the clinical efficacy of the Kim's protocol retreatment scheme also seems to reflect into a more pronounced reduction in anti-MAG antibody titer at follow-up, although not reaching statistical significance in this smaller subsample of patients. Based on current evidence, there is no definitive correlation between anti-MAG antibody titers and the clinical severity or progression of neuropathy (32). Nonetheless, anti-MAG titers may serve as an indirect marker of therapeutic response, particularly in the context of B-cell depleting therapies such as RTX, as they likely reflect suppression of the underlying monoclonal gammopathy. Therefore, while baseline titers alone do not predict disease severity, and their reduction post-treatment may suggest effective B-cell suppression, this parameter alone is not suitable for guiding therapeutic timing. In this context, the more pronounced reduction in anti-MAG titers observed with the Kim's protocol may still be clinically relevant, indicating better control of the pathogenic B-cell clone and a greater likelihood of favorable outcomes. However, CD27+ monitoring offers a more dynamic and individualized marker of memory B-cell reconstitution, allowing preemptive retreatment and potentially optimizing RTX use.

Overall, there were no notable side effects and RTX treatment was well tolerated.

Only two patients—one in each group—became non-responders. To this end, the patient included in the *Kim's protocol* cohort became non-responder after 11 years of disease, receiving a total of 7 maintenance doses, while the patient in the *relapse* cohort became non-responder after 2 years of disease and 2 full courses of RTX. Ultimately, patients in the *relapse* cohort received a cumulative dose of RTX approximately 2.5 times higher than those in the *Kim's protocol* cohort.

Moreover, it should also be noted that, in contrast to patients in the *relapse* cohort who received the full maintenance cycle following an objectifiable clinical worsening, patients in the *Kim's protocol* cohort received maintenance infusions based on the re-emergence of CD27+, independently from their clinical status, without waiting for their conditions to deteriorate.

Notably, no patients experienced clinical worsening without a concomitant increase in CD27+ memory B cells. Conversely, we cannot determine whether CD27+ reappearance can occur without clinical relapse, as patients in the *Kim's protocol* cohort were re-treated regardless of clinical status; this difference in treatment strategy precludes direct assessment of whether immunological reconstitution alone could precede clinical relapse in the absence of timely intervention.

Finally, if we consider an average time of 14.9 months between RTX infusion and the reappearance of CD27+ B lymphocytes, we can speculate that also a fixed treatment schedule every 6 months may represent overtreatment (12, 13).

These findings highlight that an individualized treatment strategy based on CD27+ monitoring is superior to a relapse-driven therapy in terms of sensory improvement, but more importantly, it allows for the administration of a lower cumulative dose of RTX while maintaining comparable safety and tolerability. This is particularly relevant in terms of cost-effectiveness, as it reduces the overall amount of drug required, and from a logistical perspective, by minimizing the number of infusions needed. These aspects are crucial not only for patients and their quality of life but also for healthcare facilities, which would benefit from reduced pharmaceutical and organizational costs associated with infusion management when adopting this treatment strategy.

From a health economics standpoint, monitoring memory B-cell reconstitution via CD27 expression on peripheral blood lymphocytes adds a modest cost (~€30–40 per test in Italy) when incorporated into standard flow cytometry panels. With quarterly testing, the estimated annual cost is ~€120–160 per patient. In our study, patients following the Kim's protocol received a mean cumulative RTX dose of 2,250 mg/m^2^, significantly lower than the 5,526 mg/m^2^ observed in the relapse cohort, corresponding to an approximate per-patient drug cost reduction of ~€12,900 (based on the Italian NHS reference price) (33). These findings suggest that CD27-guided RTX dosing could optimize treatment while significantly lowering costs, supporting its potential as a feasible and economically advantageous biomarker strategy in clinical practice.

It is essential to acknowledge some limitations. The retrospective study design and lack of randomization may introduce unmeasure confounding and selection bias. While the two groups were comparable at baseline in terms of functional clinical scores, some demographic and longitudinal differences were present. Notably, patients in the *Kim's protocol* cohort were older at disease onset and had a significantly shorter follow-up period compared to those in the *relapse* cohort due to the different enrollment time frames. These differences may have influenced outcome trajectories and must be considered when interpreting the results. Moreover, the small sample size was insufficient to support propensity score matching or adjustment, which could lead to suboptimal estimates and further reduction of statistical power. Instead, we relied on direct comparisons between groups, using consistent inclusion criteria and uniform outcome assessment across all patients. Although this approach does not eliminate the possibility of residual confounding, it reflects a real-world clinical setting and supports preliminary comparative evidence on rituximab maintenance strategies in anti-MAG neuropathy.

As previously mentioned, there are also some limitations linked to the outcome scales employed. For instance, the ISS scale is not informative on impairment of deep sensitivities, the MRC scale does not assess distal hyposthenia, and currently useful tools for evaluating neuropathic tremor are lacking. In fact, tremor improvement was evaluated based on clinical judgment by experts during neurological examinations, reflecting real-world practice but representing a suboptimal and subjective tool. Moreover, both MRC and INCAT scores may be inadequate for detecting subtle changes, particularly in anti-MAG neuropathy where progression is often slow and heterogeneous. Furthermore, neither patient-reported outcome data nor perceived quality of life (QoL) metrics were collected. Future research would benefit from adopting more sensitive and comprehensive outcome measures, including validated tremor rating scales and patient-reported outcomes such as SF-36, I-RODS, or NeuroQoL for peripheral neuropathy (34–36), to better capture the multidimensional impact of neuropathy and treatment effects.

To overcome current limitations, future studies with larger sample sizes and prospective designs—ideally randomized controlled trials—are needed to define the optimal maintenance regimen with RTX in anti-MAG polyneuropathy. In parallel, emerging therapeutic options may further enhance treatment strategies. Subcutaneous rituximab (SC-RTX), for instance, has shown comparable efficacy and safety to the intravenous formulation, while offering practical advantages such as shorter administration times and greater patient convenience, potentially reducing treatment burden (37, 38). Additionally, novel agents such as Bruton's tyrosine kinase inhibitors (BTKis) are gaining attention, particularly in cases of anti-MAG neuropathy associated with *Waldenström macroglobulinemia*, supported by preliminary evidence of efficacy (39–41). Collaborative clinical trials involving both neurologists and hematologists will be key to integrating these emerging therapies into future care paradigms.

## 5 Conclusions

Our results suggest that a tailored maintenance regimen with RTX based on the reemergence of peripheral CD27+ memory B cells may be more cost-effective by drastically lowering the cumulative drug dose and, as a result, the overall treatment burden, while also offering potential advantages in terms of clinical response when compared to treatment upon clinical relapse.

## Data Availability

The raw data supporting the conclusions of this article will be made available by the authors, without undue reservation.
